# Immune effector cell-associated enterocolitis following chimeric antigen receptor T-cell therapy in multiple myeloma

**DOI:** 10.1038/s41408-024-01167-8

**Published:** 2024-10-16

**Authors:** Gliceida Galarza Fortuna, Rahul Banerjee, Constanza Savid-Frontera, Jinming Song, Carlos M. Morán-Segura, Jonathan V. Nguyen, Lazaros Lekakis, Sebastian Fernandez-Pol, Annie N. Samraj, Kikkeri N. Naresh, Mariola Vazquez-Martinez, Rachid C. Baz, Jay Y. Spiegel, Lekha Mikkilineni, John M. Gubatan, Surbhi Sidana, Andre de Menezes Silva Corraes, Nilesh M. Kalariya, Krina K. Patel, Kevin G. Shim, Rafael Fonseca, Christopher Ferreri, Peter M. Voorhees, Shambavi Richard, Cesar Rodriguez Valdes, Sireesha Asoori, Jeffrey L. Wolf, Andrew J. Cowan, Douglas W. Sborov, Frederick L. Locke, Yi Lin, Yinghong Wang, Doris K. Hansen

**Affiliations:** 1 Huntsman Cancer Institute, University of Utah, Salt Lake City, UT USA; 2 Fred Hutchinson Cancer Center, Seattle, WA USA; 3 University of Washington, Seattle, WA USA; 4 Moffitt Cancer Center, Tampa, FL USA; 5 Sylvester Comprehensive Cancer Center, University of Miami, Miami, FL USA; 6 Stanford University, Palo Alto, CA USA; 7 Mayo Clinic, Rochester, MN USA; 8 The University of Texas MD Anderson Cancer Center, Houston, TX USA; 9 Mayo Clinic Arizona, Scottsdale, AZ USA; 10 Levine Cancer Institute, Charlotte, NC USA; 11 Mount Sinai Tisch Cancer Institute, New York, NY USA; 12 University of California San Francisco, San Francisco, CA USA

**Keywords:** Myeloma, Drug development, Cancer immunotherapy

## Abstract

We report 14 cases of immune effector cell (IEC)-associated enterocolitis following chimeric antigen receptor T-cell (CAR-T) therapy in multiple myeloma, with a 1.2% incidence overall (0.2% for idecabtagene vicleucel and 2.2% for ciltacabtagene autoleucel). Patients developed acute-onset symptoms (typically non-bloody Grade 3+ diarrhea) with negative infectious workup beginning a median of 92.5 days (range: 22–210 days) after CAR-T therapy and a median of 85 days after cytokine release syndrome resolution. Gut biopsies uniformly demonstrated inflammation, including intra-epithelial lymphocytosis and villous blunting. In one case where CAR-specific immunofluorescence stains were available, CAR T-cell presence was confirmed within the lamina propria. Systemic corticosteroids were initiated in 10 patients (71%) a median of 25.5 days following symptom onset, with symptom improvement in 40%. Subsequent infliximab or vedolizumab led to improvement in 50% and 33% of corticosteroid-refractory patients, respectively. Five patients (36%) have died from bowel perforation or treatment-emergent sepsis. In conclusion, IEC-associated enterocolitis is a distinct but rare complication of CAR-T therapy typically beginning 1–3 months after infusion. Thorough diagnostic workup is essential, including evaluation for potential T-cell malignancies. The early use of infliximab or vedolizumab may potentially hasten symptom resolution and lower reliance on high-dose corticosteroids during the post-CAR-T period.

## Introduction

Several immune effector cell (IEC) therapies targeting B-cell maturation antigen (BCMA) have been approved by the United States (US) Food and Drug Administration (FDA) for the treatment of multiple myeloma (MM), including chimeric antigen receptor T-cell (CAR-T) therapies such as idecabtagene vicleucel (ide-cel) and ciltacabtagene autoleucel (cilta-cel) [1, 2]. Common IEC-associated adverse events include cytokine release syndrome (CRS) and immune effector cell-associated neurotoxicity syndrome (ICANS), while rarer toxicities include IEC-associated hemophagocytic lymphohistiocytosis-like syndrome (IEC-HS) and late-onset neurotoxicity [3–5]. In the past five years, 3 reports of colitis manifesting as watery diarrhea have been reported following CD19-directed CAR-T therapy [6–8]. In one case, CAR T-cells expressing α4β7 integrin were found to be enriched within the colon lamina propria following treatment with tisagenlecleucel [8]. A similar case of diarrhea has recently been reported following cilta-cel in MM; however, in this case, the CAR-positive infiltrate was neoplastic consistent with T-cell lymphoma [9].

Treatment-emergent gastrointestinal (GI) toxicities following CAR-T therapy in MM have not been well characterized. Diarrhea was noted in 0.4% of patients in the ide-cel arm of KarMMa-3 (*n* = 1) and 2.4% of patients in the cilta-cel arm of CARTITUDE-4 (*n* = 5) [1, 2]; however, the details of chronology, further workup, and treatment in these six cases are unclear. Diarrhea following CAR-T therapy can potentially be attributed to other causes such as bacterial infections, clinically significant cytomegalovirus (CMV) reactivation, or organ-specific manifestations of inflammation during active CRS. Whether IEC-associated enteritis or colitis can occur in the absence of a neoplastic etiology following BCMA-directed CAR-T therapy in MM, as noted in one case following CD19-directed CAR-T therapy in lymphoma [8], is unknown.

## Methods

We performed a retrospective analysis of cases of diarrhea, enterocolitis, or colitis following commercial ide-cel or cilta-cel at 11 centers within the United States Multiple Myeloma Immunotherapy Consortium. Cases were identified by each participating center through internal discussions at standing myeloma-related and/or CAR-T related meetings for commercial infusions that may have met the above criteria. For centers with dedicated IEC compliance programs or long-term follow-up programs, these program databases were also queried. Institutional Review Board approval was obtained for participation within the Consortium at each participating center. Patients were treated according to the local standard of care at each center. Collected data points included baseline patient characteristics, CAR-T-related information, post-infusion toxicities, summaries of biopsy reports (if available), and patient outcomes. CRS and ICANS were graded using American Society for Transplantation and Cellular Therapy (ASTCT) consensus criteria, while other symptoms (diarrhea, colitis, and enterocolitis) were graded using Common Terminology Criteria for Adverse Events (CTCAE) version 5.0.

Endoscopic biopsies were reviewed by board-certified anatomic pathologists or hematopathologists at each institution. At one institution where the necessary reagents were available, multiplex immunofluorescence and immunohistochemistry (IHC) were used to analyze the presence of the following markers: CD3, CD8, CD138, and camelid V_H_H (variable heavy chain of a heavy-chain antibody) seen in ciltacabtagene autoleucel. Additional information about the methods for these assays are shown in Supplementary Table S1. For all patients, data were analyzed utilizing descriptive methods including medians, percentages, and student t-tests to compare clinical parameters in patients with resolved IEC-associated enterocolitis versus patients who died secondary to enterocolitis or associated complications. All analyses were performed using GraphPad Prism 10.2.3 (La Jolla, California, USA).

## Results

We identified 14 cases of IEC-associated enteritis and/or colitis diagnosed at one of 11 centers within the US Multiple Myeloma Immunotherapy Consortium, in one case following ide-cel and in 13 cases following cilta-cel. This corresponds to an IEC-associated enterocolitis incidence of 1.2% overall out of 1287 infusions (636 ide-cel and 651 cilta-cel) across all study centers, with product-specific incidences of 0.2% for ide-cel and 2.2% for cilta-cel. Importantly, this figure excluded 3 cases of potential or confirmed T-cell lymphoproliferative disorders, including a previously characterized patient who was managed at multiple institutions [9]; features of these 3 patients are summarized separately in Supplementary Table S2.

**Fig. 1:**
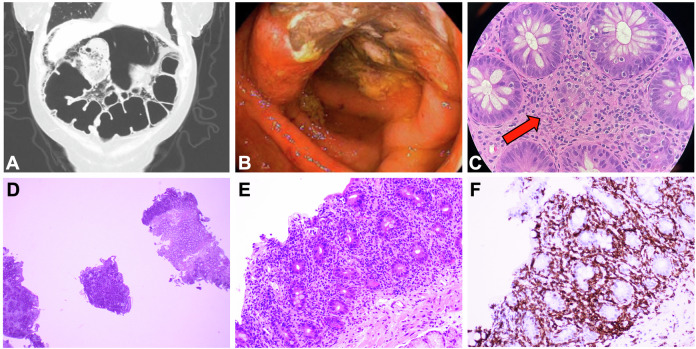
Representative images of IEC-associated enterocolitis. Top panel: Representative colitis-related images from different patients. Panel **A**: diffuse bowel wall pneumatosis and pneumoperitoneum on computed tomography imaging consistent with toxic megacolon, which subsequently required subtotal colectomy. Panel **B**: ulcerations noted on endoscopic evaluation of the transverse colon. Panel **C**: 10× magnification of colonic mucosa showing focal crypt dropout and increased apoptotic activity; minimal expansion by scattered eosinophils and focal intraepithelial lymphocytes are also seen. Bottom panel: Representative enteritis-related images from one patient. Marked infiltration of the lamina propria by T lymphocytes at low power (2×, panel **D**) and high power (10×, panel **E**). Immunohistochemical stain for CD3 (20×, panel **F**) demonstrates that the majority of the lamina propria cells and intraepithelial lymphocytes are T cells.

As detailed in Table 1, patients were evenly split between men and women and ranged in age from 39 to 79 at CAR-T infusion. One patient (7%) had a reported medical history of inflammatory bowel disease (IBD) in the distant past with no symptoms at time of infusion. Most patients (86%, *n* = 12) had previously undergone autologous stem cell transplantation (ASCT) without any post-transplantation diarrhea or engraftment syndrome. IEC-associated enterocolitis manifested as acute-onset diarrhea (non-bloody in all but one case) beginning a median of 92.5 days (range: 22–210 days) after CAR-T therapy and a median of 85 days after CRS resolution (range: 2–205 days). Diarrhea was grade 3 or higher in all cases, and 71% of patients (*n* = 10) reported concurrent abdominal pain. Computed tomography imaging (*n* = 13) showed small bowel or large bowel inflammation in 43% of cases (*n* = 6), including two cases of diffuse colonic pneumatosis (Fig. 1A). With regard to antecedent CRS and ICANS, 4 patients had maximum Grade 2 CRS, 9 maximum Grade 1 CRS, and 1 no CRS; one patient had developed Grade 2 ICANS previously. Management strategies for antecedent CRS or ICANS included tocilizumab (*n* = 11), corticosteroids (*n* = 9), and anakinra (*n* = 1). Three patients had developed delayed cranial nerve palsies following cilta-cel, with neurotoxicity resolution before enterocolitis onset in two of these cases. By the time of diarrhea onset, serum ferritin and C-reactive protein levels at symptom onset were below their peak values in all patients.

**Table 1:** Patient, disease, and enterocolitis characteristics.

Demographic characteristics at infusion	*n* (%)
Male gender	7 (50)
Age in years, median (range)	64 (39–79)
ECOG performance status, median (range)	1 (0–1)
History of inflammatory bowel disease	1 (7)
Disease and treatment characteristics	
Extramedullary disease at infusion	2 (14)
Prior lines of therapy, median (range)	4.5 (4–7)
Prior stem cell transplantation	12 (86)
CAR-T product type	
*Cilta-cel*	13 (93)
*Ide-cel*	1 (7)
Biomarkers at infusion, median (range)	
*Absolute lymphocyte count (x10*^*9*^*/L) (n* = *14)*	0.47 (0.0–3.46)
*C-reactive protein (mg/L) (n* = *13)*	2.9 (0.3–60.9)
*Ferritin (ng/mL) (n* = *13)*	120 (37–1819)
Biomarker maximum values, median (range)	
*C-reactive protein (mg/L) (n* = *13)*	19.4 (1.8–194.8)
*Ferritin (ng/mL) (n* = *13)*	420 (111–11214)
Any-grade CRS	13 (93)
CRS grade ≥2	4 (29)
Any-grade ICANS	1 (7)
ICANS grade ≥2	1 (7)
≥PR to CAR-T	10 (71)
AE characteristics at symptom onset	
Days after infusion, median (range)	92.5 (22–210)
Days after CRS resolution, median (range)	85 (2–205)
Highest CTCAE grade, median (range)	3 (1–5)
Diagnostic presentation	
*Non-bloody diarrhea*	13 (87)
*Radiographic enteritis or colitis*^a^ *(n* = *14)*	6 (43)
Biomarkers at onset, median (range)	
*Absolute lymphocyte count (x10*^*9*^*/L) (n* = *13)*	0.84 (0.12–3.02)
*C-reactive protein (mg/L) (n* = *9)*	3.30 (0.3–14.7)
*Ferritin (ng/mL) (n* = *6)*	92 (33–3462)
*IgG mg/dL (n* = *14)*	326.5 (25–778)
AE treatment and outcomes	
Systemic corticosteroid use	10 (71)
*Intravenous corticosteroids*	4 (40)
*Oral corticosteroids*	6 (60)
Duration of corticosteroids in days, median (range)	31 (1–45)
Infliximab use	6 (43)
Infliximab doses, median (range)^b^	1 (1–3)
Clinical benefit from infliximab (*n* = 6)	3 (50)
Vedolizumab use	3 (20)
Vedolizumab doses, median (range)^b^	2 (2-2)
Clinical benefit from vedolizumab (*n* = 3)	1 (33)
AE status (as of data cutoff)	
*Resolution of symptoms*	4 (28)
*Ongoing symptoms*	5 (36)
*Death due to enterocolitis*	5 (36)
Days to symptom resolution, median (range)^a^	113 (76–188)

*AE* adverse event, *CAR-T* chimeric antigen receptor T-cell therapy, *CRS* cytokine release syndrome, *CTCAE* Common Terminology Criteria for Adverse Events, *dl* deciliter, *ECOG* Eastern Cooperative Oncology Group, *ICANS* immune effector cell-associated neurotoxicity syndrome, *L* liter, *mg* milligrams, *mL* milliliter, *ng* nanograms, *PR* partial response, *IgG* immunoglobulin G.

^a^Only in patients with resolved symptoms.

^b^When administered, infliximab and vedolizumab were dosed with an interval of 2 weeks between the second dose and the first dose and an interval of 6 weeks between the third dose and the second dose.

**Fig. 2:**
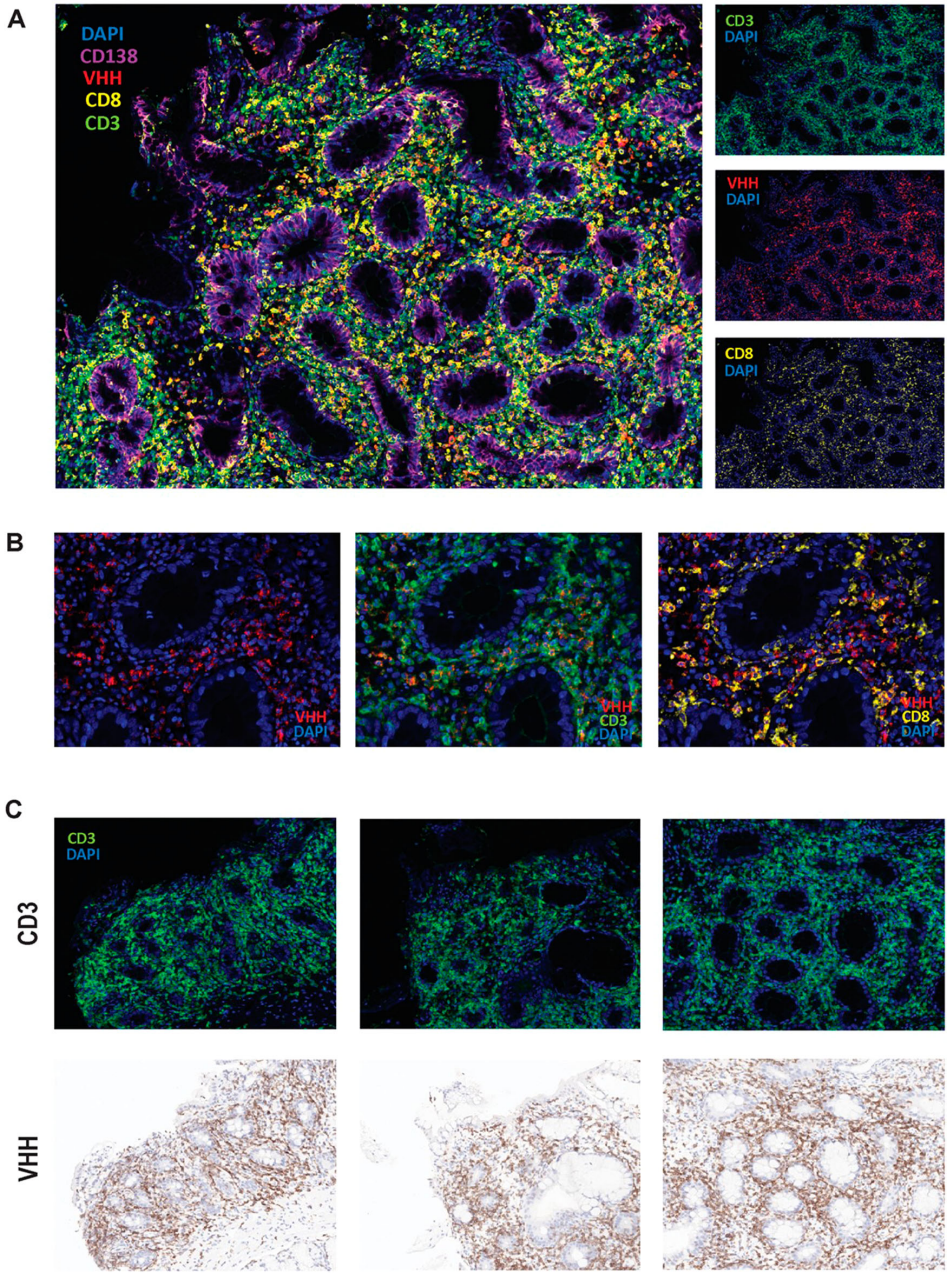
Evidence of CAR T-cell infiltration into gut lamina. Multiplex immunofluorescence (mIF) images from a duodenal FFPE biopsy of a patient who developed IEC-associated enterocolitis after ciltacabtagene autoleucel therapy for multiple myeloma. Panel **A** shows a representative merged image demonstrating presence of CAR+ (Camelid VHH+, red) T cells (CD3+, green) infiltrating the affected tissue. CD8 staining (yellow) determines CD8+ and CD8− T cell subsets among the T cells. Single channel images of the field of view are shown on the right. Panel **B** shows (1) Camelid V_H_H detection by mIF (left), (2) co-expression of CD3 and VHH (center) demonstrating that infiltrating CAR+ (red) cells are CAR-transduced CD3+ T cells (green), and (3) differential expression of CD8 and V_H_H (right) indicating that some but not all CAR-T cells are CD8+ (yellow) cells. Panel **C** shows representative images of different areas of the biopsy identifying CD3+ T cells (green) by mIF infiltrating the tissue (top). Those same regions from the IHC-stained slide are shown (bottom), demonstrating similar pattern of infiltration of CAR+ cells (V_H_H+), indicating that great majority of the infiltrating T cells are CAR-T cells. CAR chimeric antigen receptor, FFPE formalin-fixed paraffin-embedded, IEC immune effector cell, IHC Immunohistochemistry, V_H_H variable heavy chain of a heavy-chain antibody.

The results of endoscopic evaluations were available for 13 patients (93%): 6 patients underwent both esophagogastroduodenoscopy (EGD) and colonoscopy, 6 patients had only a colonoscopy, and 1 patient had only an EGD. Biopsies uniformly demonstrated evidence of inflammation in at least one area (Supplementary Table S3). Colonic biopsies (representative images in Fig. 1B, C) typically showed endoscopic evidence of ulceration with histological evidence of crypt dropout and increased apoptotic activity. Duodenal biopsies (representative images in Fig. 1D–F) typically showed villous blunting and increased intraepithelial lymphocytes. In several cases (*n* = 6), marked intra-epithelial lymphocytosis was noted. Chronic changes such as crypt architecture distortion were only rarely seen. Biopsy findings were often noted by interpreting pathologists to resemble those seen in graft-versus-host disease (GVHD); however, all patients had received autologous CAR-T therapy and no patients had previously undergone allogeneic transplantation. At one institution with access to antibody-based testing for the camelid V_H_H region found in cilta-cel (Fig. 2), multiplex immunofluorescence and IHC staining revealed CAR-transduced T cells (some CD8-positive and some CD8-negative) infiltrating into the duodenal lamina propria.

With regard to infectious workup, alternative bacterial causes of diarrhea were not identified at symptom onset. Median IgG level at symptom onset was 326.5 milligrams per deciliter (range: 25–778), and 71% (*n* = 10) of patients had received intravenous immunoglobulin within 30 days beforehand. Peripheral blood CMV assessment by quantitative polymerase chain reaction testing was performed in 11 cases: results were negative in 7 cases, below the lower level of detection in 2 cases, and frankly positive in two cases (peak values 186 and 12,800 international units per milliliter, respectively). Both patients with CMV viremia received intravenous ganciclovir with CMV clearance in one case and ongoing low-level positivity in the other. Immunohistochemical stains of enteric biopsies for bacterial and viral infections (including CMV) from biopsies were negative in all cases, including in both patients with CMV viremia.

With regard to treatment, systemic corticosteroids were initiated in 71% of cases (*n* = 10). This included six patients who received oral prednisone with a median starting dose of 0.87 milligrams (mg) per kilogram (kg) per day (range: 0.19–2 mg/kg/day) for a median duration of 21 days (range: 1–145 days) as well as four patients who received intravenous methylprednisolone with a median starting dose of 1.40 mg/kg/day (range: 1–2 mg/kg/day) for a median duration of 49 days (range: 20–68 days). Corticosteroids were initiated a median of 26 days (range: 1–80 days) following symptom onset, and 40% of steroid-treated patients had symptomatic improvement thereafter. Six patients with corticosteroid-refractory symptoms (43% of all cases) were subsequently started on infliximab based on American Society of Clinical Oncology (ASCO) guidelines for managing immune-related adverse events (irAEs) following checkpoint inhibitors [10]. Median time to infliximab initiation was 61 days (range: 34–100 days) after diarrhea onset, and half of infliximab-treated patients had symptomatic improvement after 1–3 doses of infliximab. Given that α4β7 integrin may be overexpressed on CAR T-cells within the colon [8], the α4β7 integrin antagonist vedolizumab was prescribed in three patients (21% of all patients). Vedolizumab was initiated a median of 59 days (range: 40–120) after diarrhea onset, with one of three patients having symptomatic improvement but subsequently developing *Clostridioides difficile* infection requiring fecal microbiota transplantation.

With a median follow-up of 187 days since diarrhea onset (range: 44–532 days), 4 patients have had diarrhea resolution (median time to resolution 113 days), including one patient who required a 145-day prednisone course with a taper and two patients who required 1–3 doses of infliximab concomitantly with corticosteroids. Five patients have ongoing diarrhea, including two patients with improved but ongoing symptoms. Five patients (36% of all cases) have died, either directly from bowel perforation secondary to IEC-associated enterocolitis (*n* = 3) or from treatment-emergent sepsis (*n* = 2). Characteristics of patients with symptom resolution (*n* = 4) versus deceased patients (*n* = 5) are shown in Supplementary Table S4. Albeit with small *n*, no significant differences were noted in terms of age, pertinent biomarkers, time to symptom onset, or time to corticosteroid initiation.

## Discussion

This represents the largest case series of IEC-associated enterocolitis following any type of CAR-T therapy. Clinical and histological presentations were strikingly similar in most cases, suggesting that IEC-associated enterocolitis is a rare but distinct toxicity of CAR-T therapy characterized by non-bloody diarrhea with negative infectious workup beginning 1–3 months following CAR-T therapy. Inflammation is typically seen on enteric biopsies with a specific resemblance to the pattern seen in GVHD following allogeneic transplantation. The addition of biologic immunosuppressive agents (e.g., infliximab or vedolizumab dosed at weeks 0, 2, and 6) may benefit patients with corticosteroid-refractory symptoms. Indeed, in the setting of colitis as an irAE following immune checkpoint inhibition, ASCO guidelines recommend initiating infliximab as soon as 3 days after corticosteroids if symptoms do not begin to improve [10]. Vedolizumab may also be helpful given that α4β7 integrin (the target of vedolizumab) has been shown to be overexpressed on gut-trafficking CAR T cells [8]. Other management approaches such as ruxolitinib or lymphotoxic chemotherapy are considerations in refractory IEC-HS [4] and could be considered for life-threatening cases of enterocolitis as well. Of course, further research is needed to validate the utility of any of these immunosuppressive agents in this specific scenario. Based on our collective experience to date, key recommendations for diagnosing and managing IEC-associated enterocolitis are shown in Table 2.

**Table 2:** Recommendations for diagnosing and managing IEC-associated enterocolitis.

*Diagnosing IEC-associated enterocolitis*	
Recommendation	Rationale
Consider this entity in patients with unexplained diarrhea following CAR-T, particularly if > 1 month afterward	In our series, the median time to symptom onset (typically Grade 3+ non-bloody diarrhea) was 3 months after CAR-T infusion
(If applicable) Re-refer the patient to the CAR-T treatment center if no longer being followed there actively	Diagnosis of this delayed toxicity requires close consultation between the patient’s primary oncologist and the CAR-T treatment center
Perform endoscopic evaluation with biopsies that are specifically reviewed by a hematopathologist	Infectious causes (e.g., CMV colitis) can occur without viremia, and enteral T-cell malignancies have been reported in this setting [9]
(If applicable) Work with product manufacturers to test for CAR presence on enteral biopsies	If a lymphoproliferative process is suspected, the product manufacturer will be able to assist with CAR staining to evaluate causality

*Managing IEC-associated enterocolitis*	
Recommendation	Rationale
Consult with GI and ID specialists on management and strategies to avoid treatment-related infections	Given the rarity of this toxicity and the immunosuppressive nature of its management, a collaborative approach is imperative
Consider minimizing the use of long-term corticosteroids, particularly if symptoms do not resolve quickly	Long-term corticosteroids can potentially predispose patients to complications such as adrenal insufficiency or bowel perforation
Consider irAE-type management with early infliximab or vedolizumab if symptoms do not resolve quickly	In our series, these biological agents occasionally led to symptom resolution in steroid-refractory cases after 1–3 doses
If symptoms do not resolve with the above steps, revisit the diagnosis of a potential lymphoproliferative process	For patients with lymphoproliferative T-cell processes involving the gut, drugs like cyclosporine may be more effective [12]
For life-threatening cases, consider IEC-HS management strategies including ruxolitinib or chemotherapy	While these agents have not been studied in this setting, they are options in refractory cases of IEC-HS based on expert opinion [4]

All recommendations are based on our experience to date. Further research into diagnostic modalities and therapeutic interventions, ideally studied in a prospective manner, will be important steps to advance our understanding of IEC-associated enterocolitis.

*CAR* chimeric antigen receptor, *CAR-T* chimeric antigen receptor T-cell therapy, *CMV* cytomegalovirus, *GI* gastroenterology, *ID* infectious diseases, *IEC* immune effector cell, *irAE* immune-related adverse event.

IEC-associated enterocolitis remains a rare phenomenon, with an incidence of 1.2% out of over 1200 commercial or out-of-specification BCMA CAR-T infusions across all study centers. Comparing our observed product-specific incidences of 0.2% for ide-cel and 2.2% for cilta-cel is difficult given the novelty and rarity of this toxicity. The voluntary FDA Adverse Events Reporting System (FAERS) database, which has been used previously to characterize rare toxicities of CAR-T therapy [11], includes 22 reactions that may potentially be linked to IEC-associated enterocolitis (Supplemental Table S5). However, FAERS data do not have a denominator and it is unclear whether any of these reported reactions represent infectious sequelae, pre-existing conditions, or duplicate entries (either with each other or with cases described here). The recently characterized case of CAR-positive T-cell lymphoma following cilta-cel is not isolated [9], but such cases are even rarer in our experience. Failure of enterocolitis symptoms to improve with corticosteroids and biological immunosuppressive agents should prompt consideration of a lymphoproliferative process for which, after diagnostic confirmation of neoplastic T cells, drugs such as cyclosporine may be more appropriate [12].

The mechanism of IEC-associated enterocolitis remains unclear. BCMA is expressed on GI-associated lymphoid tissue and plays a role in promoting lymphocyte class switching toward IgA production [13, 14]. However, given that BCMA expression is similar in tonsillar and enteric tissue [13] and that similar presentations have occurred with CD19-directed CAR-T therapy [6–8], direct on-target toxicity does not seem to fully explain IEC-associated enterocolitis. BCMA in the lamina propria is mostly found on long-lived IgA plasma cells, which likely modulate gut immunity and the gut microbiome [15–17]. It is thus possible that eradication of these BCMA-positive plasma cells can lead to an autoimmune-type phenomenon in a small percentage of patients. Common variable immunodeficiency (CVID), which can also manifest with gut lymphoid aggregates and an absence of small bowel plasma cells on enteric biopsies [18–20], is characterized by hypogammaglobulinemia and may occasionally present with diarrhea via a similar mechanism. However, we do not believe that CVID alone would explain our patients’ presentations given their rapid-onset symptoms and lack of CVID sequelae elsewhere. Regardless of the potential physiology, it is unclear why IEC-associated enterocolitis appears to occur in only a very small subset of patients following CAR-T therapy.

Our study has several limitations, most prominently the inability to definitively prove an association between CAR-T therapy and enterocolitis. While Fig. 2 clearly demonstrates the presence of infiltrating CAR T cells within the lamina propria of an affected patient, this does not conclusively demonstrate causality. Regardless, we believe that the timing of symptom onset and the lack of alternative etiologies both support a causal association with CAR-T therapy. The enterocolitis cases seen here are unlikely to be related to underlying MM given the relative rarity of acute-onset idiopathic diarrhea in patients who are not receiving lenalidomide or high-dose alkylating chemotherapy, which none of the patients in our analysis had recently received. While fludarabine and cyclophosphamide can potentially cause diarrhea, the timing of symptom onset (a median of 3 months after CAR-T infusion) argues against a toxicity of lymphodepleting chemotherapy. Infectious causes are unlikely given the negative workup in each case, both by stool testing and on tissue examination; however, CMV colitis can technically occur in the absence of CMV viremia or pathological identification on biopsies. While only one patient had a history of IBD in the remote past, it is possible but unlikely that an undiagnosed gut disorder such as IBD or irritable bowel syndrome may have been unmasked by the physiologic stress of CAR-T therapy. Finally, because we did not systematically evaluate all cases of diarrhea at every participating center, milder or self-limited cases may not have been reported and thus our collective incidence of 1.2% may potentially be an underestimate.

Other study limitations include the fact that not all patients had biopsy results available for review. Biopsy findings were not reviewed centrally, making it difficult to uniformly estimate histological patterns or proportions of immune cell subsets within biopsy specimens. The camelid V_H_H assay for cilta-cel is not uniformly available or validated in this setting. Finally, in contrast to 3 tentative cases of lymphoproliferative disorders presenting with diarrhea that our consortium has identified separately (Supplemental Table S1), T-cell lymphomas were not suspected in these 14 primary cases based on their morphologic and immunophenotypic features. While the stains in Fig. 2 do not definitively rule out a clonal population, the presence of both CD8-positive and CD8-negative T cells argues against a lymphoproliferative process. As boundaries between reactive lymphoid infiltrates and neoplastic lymphoid infiltrates are not always distinct, however, any suspicious infiltrate should undergo thorough evaluation by an expert hematopathologist. If a lymphoma is diagnosed, it should be reported to the FDA given the rare occurrence of T-cell lymphomas following BCMA CAR-T therapy [9, 21, 22]. Testing for the presence of CAR-positive T cells is reasonable to pursue in all cases of gut biopsies demonstrating lymphocytic infiltrates following CAR-T therapy. Nonetheless, our experience highlights the urgent need for our field to immediately recognize this entity as a unique IEC-associated toxicity. This is underscored by the fact that diarrhea persisted for almost a month before median corticosteroid initiation, in most cases well after patients had been discharged from their CAR-T treatment centers.

## Conclusion

In conclusion, IEC-associated enterocolitis is a distinct but rare complication of CAR-T therapy characterized by non-bloody diarrhea typically beginning 1–3 months after infusion. Notably, this toxicity generally occurs after patients have been discharged from their CAR-T treatment facilities. Thorough diagnostic workup, including evaluation for potential T-cell malignancies, and appropriate management with an expert gastroenterologist are essential. Further research into the pathogenesis of this toxicity is critical to understand this novel complication, in particular because over a third of patients in our cohort died due to IEC-associated enterocolitis or the immune compromise associated with its treatment. In the interim, the early use of infliximab or vedolizumab may potentially hasten symptom resolution and lower reliance on high-dose corticosteroids during the post-CAR-T period.

## Supplementary information

Supplemental material

## Data Availability

Deidentified data available upon reasonable request from the corresponding author.
