# Supplementary material for: Immune effector cell-associated enterocolitis following chimeric antigen receptor T-cell therapy in multiple myeloma
Source: Blood Cancer J. 2024 Oct 16;14(1):180. doi: 10.1038/s41408-024-01167-8 (PMC11484697; doi:10.1038/s41408-024-01167-8)
Supplement: Supplementary file 1 — Supplemental material [file 41408_2024_1167_MOESM1_ESM.docx]

Supplemental Information

[Table S1: Assays to assess for CAR presence in gut lamina propria 2](#_Toc175149876)

[Table S2: Patient, disease, and enterocolitis characteristics of patients with confirmed or suspected lymphoproliferative disorders (n = 3) 3](#_Toc175149877)

[Table S3: Clinical and pathologic characteristics of patients with IEC-associated enterocolitis 5](#_Toc175149878)

[Table S4: Characteristics of patients with resolved diarrhea versus deceased due to IEC-associated enterocolitis 7](#_Toc175149879)

[Table S5: Cases reported to FAERS database 8](#_Toc175149880)

# Table S1: Assays to assess for CAR presence in gut lamina propria

| **Fluorescent Multiplex Immunofluorescence/Immunohistochemistry (IHC)** |
| --- |
| Small bowel FFPE sample was obtained under an Institutional Review Board-approved protocol from a patient receiving ciltacabtagene autoleucel and developing IEC-associated enterocolitis post CAR-T infusion. Five-micron sections were either IHC stained using a Rabbit anti-Camelid VHH antibody (GenScript, 96A3F5, 1:150) or immunostained using the AKOAYA Biosciences OPAL TM 7-Color Automation IHC kit (Waltham, MA) on the BOND RX autostainer (Leica Biosystems, Vista, CA). The OPAL 7-color kit used TSA-conjugated to individual fluorophores to detect various targets within the multiplex assay. Sections were baked at 65°C for one hour then transferred to the BOND RX (Leica Biosystems). All subsequent steps were performed using an automated OPAL IHC procedure (AKOYA). OPAL staining of each antigen occurred as follows: heat induced epitope retrieval (HIER) was achieved with EDTA pH 9.0buffer for 20min at 95°C before the slides were blocked with AKOYA blocking buffer for 10 min. Then slides were incubated with primary antibody, Camelid V_H_H (GenScript, 96A3F5, 1:200, Dye620) at RT for 60 min followed by OPAL HRP polymer and one of the OPAL fluorophores during the final TSA step. Individual antibody complexes are stripped after each round of antigen detection. This was repeated five more times using the following antibodies; CD8 (DAKO, C8/144B, HIER-EDTA pH 9.0, 1:50, dye520), CD138 (Abcam, EPR6454, HIER-EDTA pH 9.0, 1:300, dye 570), and CD3 (DAKO, Rb poly, HIER- EDTA pH 9.0, 1:200, dye690). After the final stripping step, DAPI counterstain was applied to the multiplexed slide and was removed from BOND RX for coverslipping with ProLong Diamond Antifade Mountant (ThermoFisher Scientific). All slides were imaged with the PhenoImager 2.0 Solution scanner. |
| **Quantitative Image Analysis** |
| Multi-layer TIFF images were exported from InForm (AKOYA) and loaded into HALO (Indica Labs, New Mexico) for quantitative image analysis. A classifier was trained to identify areas of tumor, stroma or non-tissue regions. The classifier was created and tested on various images in the image set. The tissue was segmented into individual cells using the DAPI marker which stains cell nuclei. For each marker, a positivity threshold within the nucleus or cytoplasm were determined per marker based on published staining patterns and intensity for that specific antibody. After setting a positive fluorescent threshold for each staining marker, the entire image set was analyzed with the created algorithm. |
| **Specific Reagents Used** |
| \| **Reagent** \| **Lot#** \| **Product#** \| **Opal** \| **Clone** \| **Dilution** \| **Vendor** \| **AR** \| \| --- \| --- \| --- \| --- \| --- \| --- \| --- \| --- \| \| ***V_H_H*** \| S2302001-B \| A01860-200 \| 2026 \| 96A3F5 \| **1:200** \| GenScript \| **ER2** \| \| ***CD8*** \| 41627944 \| M7103 \| 8/2026 \| C8/144B \| **1:50** \| Dako \| **ER2** \| \| ***CD138*** \| 1014070-33 \| ab128936 \| 2026 \| [EPR6454] \| **1:300** \| Abcam \| **ER2** \| \| ***CD3*** \| 41584787 \| A0452 \| 4/2026 \| Rb poly \| **1:300** \| Dako \| **ER2** \|   Abbreviations: AR, antigen retrieval; CAR-T, chimeric antigen receptor T-cell; DAPI, 4',6-diamidino-2-phenylindole; ER2, Epitope Retrieval Solution 2; FFPE, formalin-fixed paraffin-embedded; HIER, heat induced epitope retrieval; IEC, immune effector cell; IHC, immunohistochemistry; TIFF, tag image file format; V_H_H, variable heavy chain of a heavy-chain antibody; TSA, Tyramide Signal Amplification. |

# Table S2: Patient, disease, and enterocolitis characteristics of patients with confirmed or suspected lymphoproliferative disorders (n = 3)

| **Demographic characteristics at infusion** | **n (%)** |
| --- | --- |
| Male gender | 1 (33) |
| Age in years, median (range) | 67 (50-71) |
| ECOG performance status, median (range) | 0 (0-2) |
| History of inflammatory bowel disease | 0 |
| **Disease and treatment characteristics** | |
| Extramedullary disease at infusion | 0 |
| Prior lines of therapy, median (range) | 6 (4-7) |
| Prior stem cell transplantation | 2 (67) |
| CAR-T product type |  |
| *Cilta-cel* | 3 (100) |
| *Ide-cel* | 0 |
| Biomarkers at infusion, median (range) | |
| *Absolute lymphocyte count (x10^9^/L)* | 0.65 (0.38-1.71) |
| *C-reactive protein (mg/L)* | 4 (0-37) |
| *Ferritin (ng/mL) (n=2)* | 489 (306-672) |
| Biomarkers at peak following infusion, median (range) | |
| *C-reactive protein (mg/L)* | 37 (4.4-263.5) |
| *Ferritin (ng/mL)* | 953 (357-1270) |
| Any-grade CRS | 2 (66) |
| CRS grade ≥ 2 | 0 |
| Any-grade ICANS | 0 |
| ≥PR to CAR-T | 3 (100) |
| **AE characteristics at symptom onset** | |
| Days after infusion, median (range) | 94 (75-122) |
| Days after CRS resolution, median (range) (n=2) | 75.5 (66-85) |
| Highest CTCAE grade, median (range) | 4 (3-4) |
| Diagnostic presentation |  |
| *Non-bloody diarrhea* | 3 (100) |
| *Radiographic enteritis or colitis^†^ (n=2)* | 2 (100) |
| Biomarkers at onset, median (range) |  |
| *Absolute lymphocyte count (x10^9^/L)* | 0.88 (0.08-2.22) |
| *C-reactive protein (mg/L) (n=1)* | 2.90 |
| *Ferritin (ng/mL) (n=1)* | 351 |
| *IgG (mg/dL) (n=2)* | 291 (153-429) |
| **AE treatment and outcomes** | |
| Systemic corticosteroid use | 3 (100) |
| Duration of corticosteroids in days, median (range) | 6 (4-100) |
| Infliximab use | 2 (67) |
| Infliximab doses, median (range)* | 2 (2-3) |
| Clinical benefit from infliximab (n = 2) | 1 (50) |
| Vedolizumab use | 1 (33) |
| Vedolizumab doses, median (range)* | NA |
| Clinical benefit from vedolizumab (n=1) | 0 |
| **AE status (as of data cutoff)** | |
| *Resolution of symptoms* | 1 (33) |
| *Ongoing symptoms* | 2 (67) |
| *Death due to colitis* | 0 (0) |
| Days to symptom resolution, median (range)^†^ | 137 |

* When administered, infliximab and vedolizumab were dosed with an interval of 2 weeks between the second dose and the first dose and an interval of 6 weeks between the third dose and the second dose.

^†^ Only in patients with resolved symptoms, in this case 1 patient whose symptoms improved rapidly after cyclosporine initiation.

Abbreviations: AE, adverse event; CAR-T, chimeric antigen receptor T-cell therapy; CRS, cytokine release syndrome; CTCAE, Common Terminology Criteria for Adverse Events; dL, deciliter; ECOG, Eastern Cooperative Oncology Group; ICANS, immune effector cell-associated neurotoxicity syndrome; L, liter; mg, milligrams; mL, milliliter; ng, nanograms; PR, partial response.

# Table S3: Clinical and pathologic characteristics of patients with IEC-associated enterocolitis

| **Patient** | **Outcome** | **Age** | **Sex** | **Product** | **Days to diarrhea onset** | **Procedure Performed** | **Summary of Representative Pathology Description** | **Treatment** |
| --- | --- | --- | --- | --- | --- | --- | --- | --- |
| 1 | Resolved | 73 | F | Ide-cel | 22 | Colonoscopy | Colonic mucosa with marked apoptosis, including apoptotic crypt abscess and focal crypt dropout. | Prednisone |
| 2 | Resolved | 63 | M | Cilta-cel | 99 | EGD | Duodenal mucosa with marked inflammation in the lamina propria consisting of lymphocytes and neutrophils with associated surface erosion, consistent with active chronic duodenitis. | Prednisone  Infliximab |
| 3 | Resolved | 57 | F | Cilta-cel | 132 | EGD and Colonoscopy | Duodenal mucosa with patchy gastric mucin cell metaplasia, mildly increased crypt apoptosis, absence of plasma cells in lamina propria, and reactive epithelial change. | Prednisone  Infliximab |
| 4 | Resolved | 76 | M | Cilta-cel | 159 | EGD and Colonoscopy | Duodenal mucosa with marked villous blunting and  increased intraepithelial lymphocytes. | Supportive |
| 5 | Improved | 77 | M | Cilta-cel | 42 | Colonoscopy | Focal minimal enteritis with crystalline material and overall preserved crypt and villous architecture. No crypt abscesses were noted. | Methylprednisolone  Infliximab |
| 6 | Improved | 66 | M | Cilta-cel | 64 | Colonoscopy | Terminal Ileum mucosa with moderate increase in eosinophils and apoptotic bodies. Colonic mucosa with slight increase in eosinophils and apoptotic bodies. | Prednisone  Infliximab |
| 7 | Ongoing | 39 | F | Cilta-cel | 71 | Colonoscopy | Mildly active chronic nonspecific enterocolitis. | Prednisone |
| 8 | Ongoing | 51 | F | Cilta-cel | 106 | EGD and Colonoscopy | Ileal mucosa with prominent denudation of epithelial cells, decreased number of Paneth cells and reactive hypermucinous changes. The lamina propria shows lymphocytic infiltrates and mixed with scattered eosinophils. | Vedolizumab |
| 9 | Ongoing | 64 | F | Cilta-cel | 125 | EGD and Colonoscopy | Duodenal mucosa with patchy active duodenitis with dense intraepithelial lymphocytes, variable villous blunting, gastric foveolar metaplasia, and increased crypt apoptosis with crypt injury and patchy active colitis with limited mildly increased crypt apoptosis. | Supportive |
| 10 | Deceased | 58 | F | Cilta-cel | 46 | Colonoscopy | Duodenal mucosa with foveolar metaplasia, mild atrophy, increased intraepithelial apoptotic bodies, patchy active duodenitis, and associated reactive epithelial changes. Colonic mucosa with patchy active colitis, mild increase in intraepithelial apoptotic bodies, and associated reactive epithelial changes. | Methylprednisolone  Vedolizumab |
| 11 | Deceased | 79 | M | Cilta-cel | 79 | Colonoscopy | Small intestinal mucosa with prominent crypt epithelial apoptosis and surface epithelial injury. Striking crypt epithelial apoptosis, as well as a prominent surface epithelial injury with associated villous attenuation in addition to active colitis, cryptitis, and crypt abscess formation. | Methylprednisolone  Infliximab  Vedolizumab |
| 12 | Deceased | 64 | M | Cilta-cel | 86 | EGD and Colonscopy | Superficially eroded duodenal mucosa with marked infiltration of lymphocytes, more prominently in the lamina propria, duodenitis, and regenerative changes. | Methylprednisolone  Infliximab |
| 13 | Deceased | 71 | F | Cilta-cel | 104 | *Unknown* | *Outside hospital biopsy not available for review.* | Supportive |
| 14 | Deceased | 63 | F | Cilta-cel | 210 | EGD and Colonoscopy | Small intestinal mucosa with moderate to severe villous flattening, intraepithelial lymphocytosis, and prominent apoptotic activity and cell dropout. The lymphocytes that are intimately associated with crypt epithelial cells and enterocytes on the surface epithelium are only CD8+. Lymphocytes in the lamina propria are both CD4+ and CD8+ in a relatively equal ratio and distribution. | Prednisone |

Abbreviations: cilta-cel, ciltacabtagene autoleucel; F, female; ide-cel, idecabtagene vicleucel; IEC, immune effector cell; M, male.

# Table S4: Characteristics of patients with resolved diarrhea versus deceased due to IEC-associated enterocolitis

|  | **Resolved (*n* = 4)** | **Deceased**  **(*n* = 5)** | **P-value** |
| --- | --- | --- | --- |
| Age at infusion (years) | 67.25 | 67 | 0.97 |
| Body mass index at infusion (kg/m^2^) | 18.8 | 28.52 | 0.09 |
| Time from infusion to symptom onset (days) | 103 | 105 | 0.96 |
| ALC at symptom onset (10^9^ cells/L) | 0.38 | 0.80 | 0.16 |
| CRP at symptom onset (mg/L) | 5.45 | 1.66 | 0.71 |
| Ferritin at symptom onset (ng/mL) | 83 | 123.67 | 0.67 |
| IgG | 286.5 | 402.2 | 0.51 |
| Time from symptom onset to steroid initiation (days) | 33.67 | 17 | 0.11 |

Means are shown unless otherwise stated; missing data were omitted. P-values represent the results of two-tailed independent sample student *t* tests.

Abbreviations: ALC, absolute lymphocyte count; CRP, C-reactive protein; IEC, immune effector cell; kg/m^2^, kilograms per square meter; mg/L, milligrams per liter; ng/mL, nanograms per milliliter.

# Table S5: Cases reported to FAERS database

|  | **Ide-cel** | **Cilta-cel** | **Total** |
| --- | --- | --- | --- |
| Colitis | 0 | 9 | 9 |
| Immune-Mediated Enterocolitis | 0 | 5 | 5 |
| Large Intestine Perforation | 0 | 3 | 3 |
| Enterocolitis | 0 | 2 | 2 |
| Autoimmune Enteropathy | 0 | 1 | 1 |
| Crohn’s Disease | 0 | 1 | 1 |
| Intestinal perforation | 1 | 0 | 1 |
| **Total** | **1** | **21** | **22** |

Query performed September 30, 2024 using the FAERS database, available at <https://www.fda.gov/drugs/questions-and-answers-fdas-adverse-event-reporting-system-faers/fda-adverse-event-reporting-system-faers-public-dashboard>. Reactions were chosen from the “Gastrointestinal Disorders” reaction group if they included the terms *colitis*, *enterocolitis*, *enteropathy*, or *perforation*. The reaction *Crohn’s Disease* was added on manual review, while *Neutropenic Colitis* was removed on manual review. The generic term *Diarrhoea* was reported in 41 cases with ide-cel and 21 cases with cilta-cel; however, this was not included given its non-specific nature. It is unclear whether any of these cases represent infectious etiologies, recurrence of pre-existing inflammatory bowel disease, or duplicate entries.

Abbreviations: Cilta-cel, ciltacabtagene autoleucel; FAERS, Food and Drug Administration (FDA) Adverse Events Reporting System; ide-cel, idecabtagene vicleucel.
